# Skiers' footprint on wildlife stress: Tracing the impact of winter sports on black grouse with interactive mapping

**DOI:** 10.1002/eap.70336

**Published:** 2026-10-21

**Authors:** Luca Nelli, Lisa Marshall, Michele Battoraro, Geremia Illarietti, Alberto Meriggi, Bruno Bassano, Jason Matthiopoulos, Steve Smith, Rupert Palme

**Affiliations:** ^1^ School of Biodiversity, One Health and Veterinary Medicine University of Glasgow Glasgow UK; ^2^ Department of Earth and Environmental Sciences University of Pavia Pavia Italy; ^3^ Alpine Wildlife Research Centre, Gran Paradiso National Park Noasca (TO) Italy; ^4^ Department of Interdisciplinary Life Sciences, Konrad‐Lorenz‐Institute of Ethology University of Veterinary Medicine Vienna Austria; ^5^ Experimental Endocrinology, Department of Biological Sciences and Pathobiology University of Veterinary Medicine Vienna Austria

**Keywords:** conservation strategies, fecal glucocorticoid metabolites, interactive mapping, skiing impact, wildlife stress, winter sports

## Abstract

Human recreation in mountainous ecosystems, particularly skiing, poses a significant challenge for wildlife conservation, as evidenced by physiological stress responses observed in black grouse (*Lyrurus tetrix*). Here, we present a reusable workflow that integrates noninvasive fecal corticosterone metabolite (FCM) measurements, mixed‐effects modeling, spatial prediction, and an interactive web application to quantify and map stress responses to winter sports. We analyzed 235 fecal samples for corticosterone metabolites as indicators of stress. Using a mixed‐effects model, we observed that FCM levels in black grouse increased with higher skier numbers, particularly in proximity to ski slopes. To enhance understanding and management of these impacts, we then used the fitted model to produce 10‐m resolution stress maps under alternative scenarios and embedded these in an interactive mapping tool, hosted at http://boydorr.gla.ac.uk/lucanelli/blackgrouse_stress, to visualize the stress impacts on black grouse under different skiing and environmental scenarios. This tool supports scenario‐based management and research into wildlife disturbances and could inform future educational efforts to raise awareness among ski resort users about their potential impact on wildlife. The workflow can also be transferred to other species and disturbance contexts. Our study contributes to the broader efforts to manage human–wildlife conflicts in sensitive ecosystems, offering a novel and practical approach to harmonizing recreational activities with wildlife conservation.

## INTRODUCTION

Mountain ecosystems are renowned for their unique biodiversity, offering habitats to a myriad of species adapted to the harsh, variable conditions characteristic of high altitudes (Antonelli et al., 2018). However, these fragile ecosystems face unprecedented threats from climate change (Pearce‐Higgins & Martin, 2023), which is altering temperature regimes (Barredo et al., 2020), snow cover patterns (Dirnböck et al., 2011; Rumpf et al., 2022), and ultimately, the distribution and survival of many species (Dirnböck et al., 2011). Compounding these challenges are the effects of anthropogenic activities (Caprio et al., 2023)—ranging from habitat fragmentation due to recreational development (Tsuyuzaki, 2002; Urbanska, 1997) to direct disturbances from outdoor activities such as skiing and hiking (Sato et al., 2013; Thiel et al., 2011). These anthropogenic pressures not only exacerbate the impacts of climate change (Roseo et al., 2025) but also pose immediate threats to the health and resilience of mountain wildlife populations (Arlettaz et al., 2015). In this context, understanding the cumulative effects of these stressors on wildlife is critical for devising effective conservation strategies.

The black grouse (*Lyrurus tetrix*), with its sensitivity to environmental and human‐induced changes (Ludwig et al., 2006), is an ideal case study for investigating conservation challenges. The significant declines in its population and habitat range across Europe have drawn attention from both conservationists and researchers (Birdlife International, 2015). The species is listed by the International Union for the Conservation of Nature (IUCN) and protected under the European Directive 2009/147/EC, necessitating member states to establish special protection areas (SPAs) to promote its conservation (European Commission, 2009). Despite its protected status, the black grouse faces numerous threats, including habitat degradation, loss and fragmentation, overhunting (Ludwig et al., 2006; Storch, 2007), and notably, the disturbance from recreational activities such as skiing, which have intensified in recent decades (Caprio et al., 2023; Patthey et al., 2012; Sato et al., 2013).

In winter, populations of black grouse in the Alps spend approximately 80% of their time inside temporary snow burrows and generally will dig and roost in a fresh burrow twice daily following periods of foraging (Arlettaz et al., 2007). They also occupy these snow burrows nocturnally, though some birds will still occupy open‐air roosts, either in open habitat or in areas where there is a greater abundance of bushes and trees such as the common juniper (*Juniperus communis*) and green alder (*Alnus alnobetula*) (Bocca et al., 2014). Winter survival of black grouse may depend upon the snow burrows providing protection from predators and thermos‐insulative properties (Bocca et al., 2014). Recent intensification of ski tourism in the European Alps is thought to be increasing the anthropogenic pressure on black grouse locally (Arlettaz et al., 2007, 2015; Patthey et al., 2012). Human disturbance can flush the birds from their snow burrows, increasing their need to thermoregulate at a greater energetic cost to the bird, especially in lower ambient temperatures. Additionally, this flushing process makes them more conspicuous to predators (Arlettaz et al., 2007).

The stress response mechanism, involving the activation of the hypothalamic–pituitary–adrenal (HPA) axis and subsequent release of glucocorticoids, plays a crucial role in an animal's ability to cope with adverse conditions (Möstl & Palme, 2002; Sapolsky et al., 2000). However, chronic stress can lead to detrimental effects on health, reproduction, and survival (Wingfield et al., 2015), making the study of stress responses in black grouse particularly relevant to their conservation.

Noninvasive methods for monitoring stress, such as the analysis of fecal corticosterone metabolites (FCMs), have been highlighted as preferable for studying endangered, free‐ranging species like the black grouse, to avoid the additional stress of capture and handling (Baltic et al., 2005; Cook et al., 2000). This approach allows for the assessment of glucocorticoid levels over time, providing a comprehensive picture of the stress landscape faced by these birds.

While studies have begun to explore the impact of ski tourism, noting behavioral adaptations and elevated stress markers in areas of high recreational activity (Formenti et al., 2015), there remains a gap in our understanding of the interactions between black grouse stress responses and the complex mosaic of anthropogenic and environmental variables present in mountain habitats (Formenti et al., 2015; Patthey et al., 2012).

In addition to these environmental challenges, the lack of tourism awareness regarding environmental impacts is a pivotal concern in conservation efforts. Research has revealed a widespread lack of awareness among recreationists about their negative impact on wildlife, despite significant studies into the ecological effects of human activities (Gruas et al., 2020). The escalating impact of nature‐based tourism on wildlife underscores the urgent need for improved management and awareness in protected areas (Wójcicki et al., 2023). Incorporating social science into ecological research can offer more complete insights into visitor behavior and its impact on wildlife, enhancing our understanding of how to mitigate these effects (Bielański et al., 2018). Moreover, understanding the attitudes and perceptions of recreationists is crucial, as witnessing direct evidence of disturbance and shifting attitudes are key to increasing compliance with conservation measures (Gruas et al., 2023).

Recognizing the need to address both ecological impacts and public awareness, this study aims to bridge the existing knowledge gaps by developing and demonstrating a generalizable workflow for linking winter recreation, physiological stress, and spatial decision support. Specifically, we:

1. Develop a reproducible workflow that integrates noninvasive FCM sampling, environmental and anthropogenic covariates, mixed‐effects modeling, and scenario‐based spatial prediction to map wildlife stress in winter sports landscapes.
2. Apply this workflow to a black grouse population in an alpine ski resort to identify how anthropogenic (skier activity, proximity to ski infrastructure) and environmental (snow conditions, air temperature, elevation, slope, dominant habitats) variables influence stress level in black grouse and how these factors interact with each other.
3. Generate spatial predictions of black grouse stress levels throughout our study area, informed by these factors and highlighting expected gradients (e.g., decreasing with distance from ski runs) and potential hotspots, and embed them in an interactive web tool to visually demonstrate to tourists the potential impact of their activities on black grouse stress levels under real‐time environmental conditions and skier presence.

## METHODS

### Case study

This study was conducted within an approximately 4 km^2^ area surrounding the Chanavey Ski Resort in the Rhêmes Valley, located in the Gran Paradiso National Park (GPNP), Italy (Figure 1). The area, characterized by northwest‐facing slopes ranging from 1680 to 2160 m above sea level (asl), consists of four managed ski slopes accessible by a single chairlift and numerous marked trails. The region is primarily covered by mixed coniferous forests, predominantly European larch (*Larix decidua*) and European spruce (*Picea abies*), transitioning to open alpine grasslands and shrubbery at higher altitudes, providing a highly suitable habitat for black grouse (Bocca et al., 2014; Nelli et al., 2016; Patthey et al., 2012; Schweiger et al., 2012).

**FIGURE 1:**
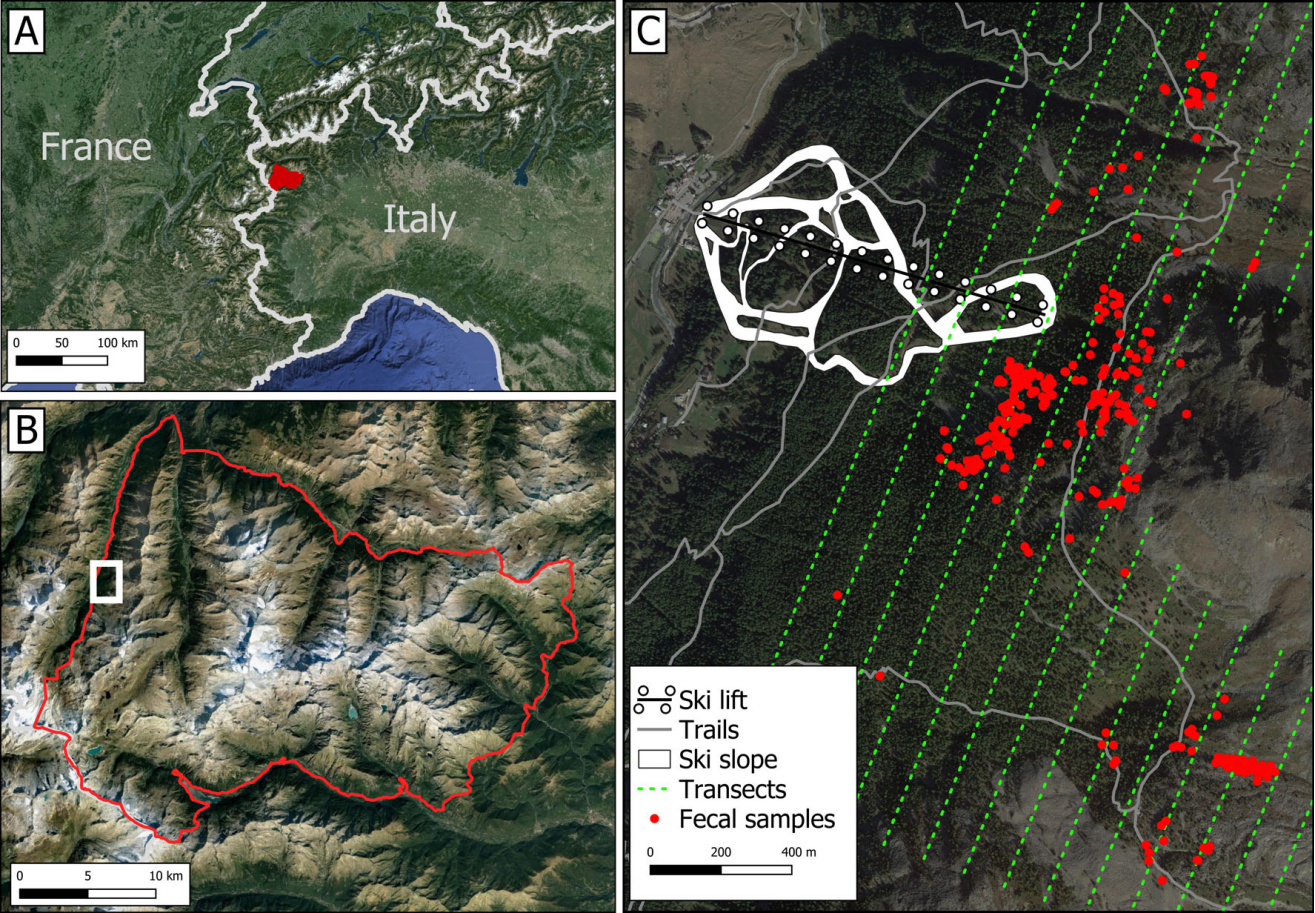
(A) Location of Gran Paradiso National Park in Italy. (B) Location of Chanavey Ski Resort in Gran Paradiso National Park. (C) Map of the ski lift facility, ski slope, transects for systematic sampling, and distribution of black grouse fecal samples. Basemap: Google Maps (https://www.google.com/maps).

### Field sampling and fecal data collection

Fecal samples were collected in the field from 7 December 2014 to 23 March 2015, aligning with the core ski season to capture the peak of recreational activity in the area. This period also corresponds to the black grouse's wintering season, a critical time for the species' survival. To ensure homogeneous coverage of the study area, parallel transects spaced 120 m apart were established and traversed at regular intervals, with the aid of touring skis or snowshoes. Each transect was walked by teams of two researchers equipped with GPS devices to record sample locations. Due to the challenging terrain, environmental conditions, and particularly harsh winter weather (intense cold and heavy snowfall), it was not always possible to strictly adhere to the predetermined paths. Deviations were made as necessary, but efforts were made to not stray more than 100 m from the original course. Within each transect, we systematically collected samples from every snow burrow visible from the path. Additionally, if fresh tracks of grouse footprints were observed, we followed these tracks until locating the associated snow burrow to collect fecal samples.

Sample collection was consistently conducted the morning after each new snowfall, allowing for a relatively accurate determination of the deposition day of the feces, as new snowfall would cover all previously deposited feces, effectively marking the day of deposition and ensuring that pellets were collected within ≤24 h of deposition (inferred from fresh snow burrows visible that morning). Moreover, the specific conditions within the snow burrows where black grouse spend the central hours of the day and the entire night prevent bacterial and enzymatic degradation of hormones and their metabolites (Arlettaz et al., 2007; Baltic et al., 2005), a crucial aspect for preserving the integrity of the FCMs for analysis. Thiel et al. (2005) have demonstrated that FCM levels measured can be considered reliable if the samples are collected within 7 days of deposition in environments where the temperature does not exceed 9°C, a condition that was well met in our study.

To minimize disturbance to the black grouse and acknowledging their behavior of spending time inside snow burrows for significant portions of the day and night, transects were walked during the early hours of the morning. This schedule aligned with the times when the birds were most likely to be foraging outside of their snow burrows. To avoid potential human contamination of the DNA and the hormonal levels in the fecal samples, gloves were worn during collection. The samples were then placed in bags, marked with an alphanumeric code for identification, and georeferenced using a GPS device.

The fecal samples were promptly stored in a chest freezer at a temperature of −20°C within 4 h from collection. This step is critical to prevent the thawing of the samples, which were already frozen due to being collected from the snow, and thus avoiding potential enzymatic degradation of the FCMs by fecal bacteria that could compromise the validity of subsequent analyses.

### Analysis of fecal corticosterone metabolites

To ensure the samples remained frozen during transit to the laboratory, they were packed in dry ice within a thermal container. In the laboratory, the samples were thawed and manually homogenized using a mechanical pestle. This step was important for the black grouse fecal samples due to the observed high variability of FCM levels within individual pellets of the same excretion (Arlettaz et al., 2007). After homogenization, the samples were dried to a constant mass at 95°C to standardize the moisture content across samples, which naturally vary in water concentration due to contamination with snow. Drying at this temperature for a short, standardized period is commonly used in fecal glucocorticoid protocols and does not degrade steroid metabolites (Baltic et al., 2005; Palme et al., 2013; Rettenbacher et al., 2004; Thiel et al., 2005). From the dried material, a subsample of 0.5 g feces was then mixed with 5 mL of a 60% methanol solution, facilitating the extraction of FCMs from the fecal matrix (Cook et al., 2000; Palme et al., 2013).

Following a 30‐min vortexing step, the samples were centrifuged, allowing for the collection of the supernatant from each sample. FCMs within the supernatant were then quantified using a cortisone enzyme immunoassay (EIA). This assay has been specifically validated for black grouse (Baltic et al., 2005) and follows the methodology outlined by Rettenbacher et al. (2004).

### Genotyping individuals

The remaining dry materials were ground to a fine powder using a mortar and pestle. Total genomic DNA was extracted from 100 mg of powdered fecal material using the FastStool kit (Qiagen, Germany) according to the manufacturer's instructions but with some slight modifications. Specifically, the incubation with Buffer AL was performed at 95°C (rather than at 70°C) for 10 min, and the final column elution step was carried out in 2 × 20 μL volumes for 10 min each. Extracts were stored at −20°C until required for downstream analyses.

Multilocus genotypes were obtained from a panel of 12 microsatellite loci run in three multiplex sets of four loci each (see Table 1 for microsatellite details). The volume per reaction was 12.5 μL, containing 6.25 μL QIAGEN Multiplex PCR Mix (Qiagen, Hilden, Germany), 160 nM of each fluorescent labeled forward primer, 160 nM of each reverse primer, and 10 ng template DNA. We performed touchdown PCR reactions to increase the specificity of the amplicons (see Weber et al., 2018 for further details). PCR products were mixed with Hi‐Di Formamide and ROX size standard (DeWoody et al., 2004) (1 μL PCR product, 8.5 μL Hi‐Di formamide, 0.5 μL ROX size standard). After a denaturation step of 5 min (95°C) and an immediate cooling on ice, PCR products were electrophoresed on an ABI 3130xl Genetic Analyzer (Applied Biosystems, Foster City, CA, USA). Alleles were scored automatically using GeneMarker ver. 2.6.0 (SoftGenetics, State College, PA, USA) and confirmed manually. In case of scoring errors or inconclusive peak patterns, samples were repeated. Samples that consistently failed DNA amplification were excluded from further analysis.

**TABLE 1:** Microsatellite loci used for genotyping black grouse fecal samples.

Locus	Na	*H*_O_	*H*_E_
TUD6^a^	5	0.47	0.54
TUT4^a^	7	0.82	0.75
TTT3^b^	9	0.93	0.84
TUT1^a^	6	0.40	0.76
BG20^c^	6	0.24	0.70
BG16^c^	7	0.61	0.69
TTD5^b^	11	0.81	0.84
BG12^c^	8	0.37	0.81
TTD1^b^	9	0.71	0.80
TTD2^b^	6	0.61	0.57
BG10^c^	10	0.38	0.78
TUT2^a^	2	0.023	0.023

*Note*: All metrics are reported per locus; no across‐locus or per‐individual averages are presented.

Abbreviations: *H*_E_, expected heterozygosity; *H*_O_, observed heterozygosity; Na, number of alleles.

^a^ DeWoody et al. (2004).

^b^ Segelbacher et al. (2000).

^c^ Piertney and Höglund (2001).

### Statistical model and scenario prediction

FCM levels, expressed in nanograms per gram, were initially normalized using a logarithmic transformation (base 10) to address their skewed distribution and make the data more amenable to the subsequent analysis.

To assess the impact of skiing activity on the black grouse, we fit a Gaussian generalized linear mixed‐effects model (GLMM), with log‐transformed FCM as response variable. As covariates, we calculated for each sample the distance from the nearest section of the ski slope, and from the nearest marked trail, which we digitized via interpretation of Google satellite imagery (https://www.google.com/maps). Using a digital land cover map (Corine Land Cover; Büttner & Kosztra, 2018), we calculated the distance between each sample and the nearest patch of each habitat category (namely, shrubs, larch forest, spruce forest, grassland, and rocks). Using a 10‐m resolution digital elevation model (Tarquini et al., 2023), we calculated the altitude and the slope of the sampling point. We also obtained meteorological variables from the nearest weather station (overseen by ARPA—the regional environment protection agency) located at an altitude of 2200 m asl, approximately 4 km from the study area, serving as an approximation of local conditions. Given that fecal information corresponds to a period of 18–24 h prior to sample discovery (Arlettaz et al., 2007), we extracted the following variables for the day prior to sampling: average temperature (in degrees Celsius), total snowfall (in centimeters), average ground snow (in centimeters). Finally, we obtained a measure of human disturbance as the daily number of passages through the chair‐lift access gate, recorded by the resort's automated entry sensor and provided by Chanavey Ski Resort. To match the approximately 18–24 h integration of FCMs, we used the previous day's total for each sampling date. Counts were used as observed (no modeling or interpolation) and treated as a proxy for on‐mountain skier presence concentrated around the lift and slope. The full list of the covariates can be found in Table 2.

**TABLE 2:** List and details of the anthropogenic and environmental explanatory variables that were included as main effects in the initial black grouse stress statistical model.

Variable	Description	Source
D_slope	Distance from sample to nearest section of slope (in meters)	Digitized by the authors
D_trail	Distance from sample to nearest section of marked trail (in meters)	
D_shrubs	Distance from sample to nearest area of shrubbery (in meters)	Corine Land Cover (10 m resolution) (https://land.copernicus.eu/en/products/clc‐backbone/clc‐backbone‐2018)
D_larch	Distance from sample to nearest larch forest (in meters)	
D_spruce	Distance from sample to nearest spruce forest (in meters)	
D_grassland	Distance from sample to nearest grassland (in meters)	
D_rocks	Distance from sample to nearest rocky area (in meters)	
ALT	Average altitude above sea level in sampling point (in meters)	10‐m digital elevation model (http://tinitaly.pi.ingv.it/)
SLOPE	Average slope in sampling point (in degrees)	
FSNOW	Total fresh snow on day prior to sampling (in centimeters)	ARPA “Lago Agnel” weather station (https://webgis.arpa.piemonte.it/meteopiemonte/)
GSNOW	Average ground snow on day prior to sampling (in centimeters)	
TEMP	Average temperature on day prior to sampling (in degrees Celsius)	
N_SKI	No. passages through the ski lift access gate (previous day)	Courtesy of Chanavey Ski Resort

We first fitted a single global GLMM with log_10_ FCM as the response. Fixed effects comprised all anthropogenic, habitat, topographic, and meteorological covariates listed in Table 2 (e.g., distance to slope/trail, distances to habitat types, altitude, slope, fresh and ground snow, temperature, and number of skiers). Because multiple fecal samples were collected from some individuals, we retained all samples and modeled the resulting repeated measures by including individual genetic ID as a random intercept in the GLMM, thereby accounting for within‐individual nonindependence and interindividual variation in FCM response (Baltic et al., 2005; Coppes et al., 2018).

We standardized all the variables by subtracting the mean and dividing the result by the SD to compare the effect size of the model coefficients. We assessed multicollinearity among independent predictors via variance inflation factors (VIFs), starting with a saturated model and sequentially excluding the variable with the highest VIF score. This process continued until all variables in the global model satisfied a moderately conservative threshold of VIF ≤6 (Rogerson, 2001), ensuring minimal multicollinearity. Qualifying variables were then evaluated for inclusion in the final model using a backward stepwise approach based on Akaike information criterion (AIC) values to refine the model to the most informative set of predictors. A priori interactions were specified between number of skiers and: distance to slope, distance to trail, altitude, ground snow, fresh snow, and distance to larch. Model refinement used backward AIC selection. Interactions were retained when they improved model fit by difference in AIC between models (ΔAIC) ≥2 and remained ecologically interpretable; when an interaction was retained, the corresponding lower order main effects were kept to preserve model hierarchy. Otherwise, terms were removed. ΔAIC values supporting the retained interaction terms are reported in Appendix S1: Table S1. We also performed 10‐fold cross‐validation, grouping samples by individual genetic ID, and compared the final AIC‐selected model with simpler alternatives (an additive‐only model without interactions and broader global specifications). Predictive performance was summarized using root mean squared error (RMSE), mean absolute error (MAE), and predictive *R* ^2^ (variance explained on held‐out folds).

Following model selection, we created a 10‐m resolution grid to comprehensively cover the study area, calculated the same environmental variables retained by the optimal model for each grid point, and we used the model to predict the expected FCM levels throughout the landscape. Given the diversity in the nature of the predictors—some being static, like distance from land cover features, and others dynamic, such as meteorological conditions and skier numbers—we generated different predictions allowing for the simulation of a variety of scenarios.

The model fitting was carried out in the statistical environment R version 4.4.1 (R Development Core Team, 2024), using the *lme4* (Bates et al., 2015) package. Spatial data handling was performed using the *sf* (Pebesma, 2018) and *terra* (Hijmans, 2020) packages.

### Interactive mapping implementation

To demonstrate the applicability of our model in assessing the impact of ski resort activity amidst different usage and meteorological scenarios, we developed an interactive mapping tool. This tool was developed using the *leaflet* (Cheng et al., 2018) and *shiny* (Chang et al., 2018) packages in *R* and ensuring color blindness friendly visualizations using the *ColorQuest* software (Nelli, 2024). It features a user‐friendly, dynamic graphical user interface (GUI), which enables the adjustment of model inputs—including skier activity levels and environmental conditions—in real time and allows us to generate and update stress risk maps for black grouse under custom scenarios.

## RESULTS

Overall, we collected 235 fecal samples (Figure 1C) from which genetic analyses identified a total of 73 distinct individuals (mean samples per individual = 3.1; SD = 2.71). Sampling was conducted across the study area, including the immediate vicinity of the ski lift and upper ski slopes, with approximately 17% of the samples collected before the opening season.

Three main effect variables and three interactions with number of skiers were selected in the top model (Table 3). The SD for the individual intercepts was 0.103, indicating variation in baseline stress levels across the population. The residual SD was 0.266, representing the unexplained variability in stress levels after accounting for the fixed and random effects. The model's marginal *R* ^2^ was 0.196, suggesting that approximately 19% of the variation in stress levels can be attributed to the measured environmental and anthropogenic factors. The conditional *R* ^2^ was 0.301, indicating that when both fixed effects and individual variability are considered, the model explains about 30.1% of the variability in stress responses among black grouse. This highlights the significant role of individual differences among black grouse in their stress response. Grouped 10‐fold cross‐validation (by individual genetic ID) supported the AIC‐selected, interaction‐inclusive model over the additive‐only alternative (RMSE 0.278 vs. 0.284; MAE 0.215 vs. 0.217; *R* ^2^ 0.085 vs. 0.063).

**TABLE 3:** Summary of the generalized linear mixed‐effects model assessing the influence of environmental and anthropogenic factors on fecal corticosterone metabolite (FCM) concentrations in black grouse.

Variables	β	SE	LCI	UCI	*p*	SD
Fixed effects						
(Intercept)	2.844	0.029	2.789	2.900	<0.001***	
N_SKI	0.066	0.033	0.001	0.130	0.043*	
GSNOW	−0.048	0.040	−0.125	0.029	0.236	
TEMP	−0.052	0.028	−0.105	0.001	0.061	
D_slope	−0.148	0.071	−0.287	−0.011	0.039*	
D_trail	0.037	0.033	−0.027	0.101	0.269	
D_larch	−0.033	0.064	−0.155	0.090	0.608	
ALT	0.162	0.071	0.025	0.300	0.024*	
N_SKI × GSNOW	−0.176	0.056	−0.282	−0.069	0.001**	
N_SKI × D_slope	−0.187	0.078	−0.335	−0.037	0.016*	
N_SKI × D_larch	−0.093	0.065	−0.218	0.032	0.152	
N_SKI × ALT	0.104	0.075	−0.041	0.246	0.165	
Random effects						
ID						0.103
Residuals						0.266

*Note*: The table lists the fixed effects and interactions retained in the final model after selection (see Table 2 for the full candidate set). Marginal *R* ^2^ = 0.196; conditional *R* ^2^ = 0.301. Significance codes: ***p < 0.001; **p < 0.01; *p < 0.1.﻿

Abbreviations: β, standardized coefficients; LCI, lower confidence interval; UCI, upper confidence interval.

A key finding from our analysis is the number of skiers on FCM levels (Figure 2A), with higher numbers associated with higher concentrations of FCMs. We also found a negative effect of the distance from the ski slope, with samples collected farther away from the slope showing lower FCM levels (Figure 2B). Altitude resulted in an important factor as well, with higher altitudes correlating with increased FCM (Figure 2C).

**FIGURE 2:**
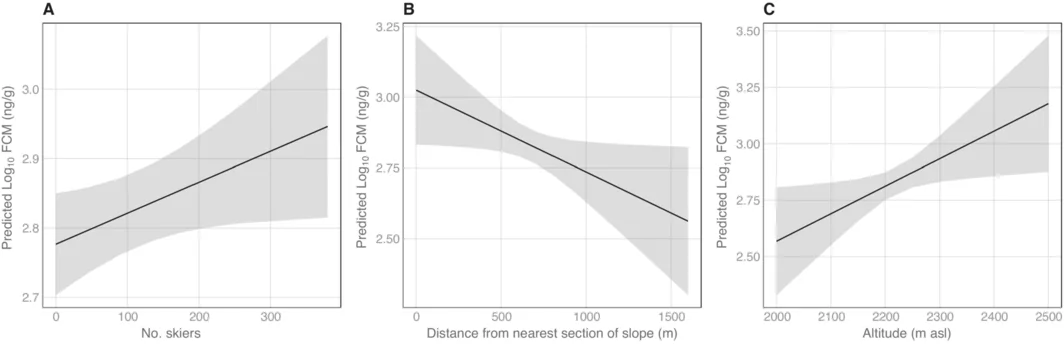
Effect plots of the relationship between various environmental factors and fecal corticosterone metabolite (FCM) levels in black grouse. (A) Number of skiers. (B) Distance from the ski slope. (C) Altitude. CIs displayed are 95% CI. Across the fitted relationships shown, predicted log_10_ FCM varied by approximately 0.17 units across the skier‐number gradient, 0.44 units across the distance‐to‐slope gradient, and 0.61 units across the altitude gradient, corresponding to approximately 1.5‐, 2.8‐, and 4‐fold differences on the original FCM scale, respectively. asl, above sea level.

The model revealed significant interaction effects between the number of skiers and the average ground snow on FCM levels in black grouse, indicating that higher levels of ground snow cover mitigate the impact of skier activity on stress levels (Figure 3A). Furthermore, a significant negative interaction effect was observed between the number of skiers and distance from the sample to the nearest section of the slope, suggesting that the effect of skier activity diminishes with increasing distance from the ski slope (Figure 3B). Additionally, the interaction between the number of skiers and average altitude was significant, with FCM levels varying across different altitudes (Figure 3C).

**FIGURE 3:**
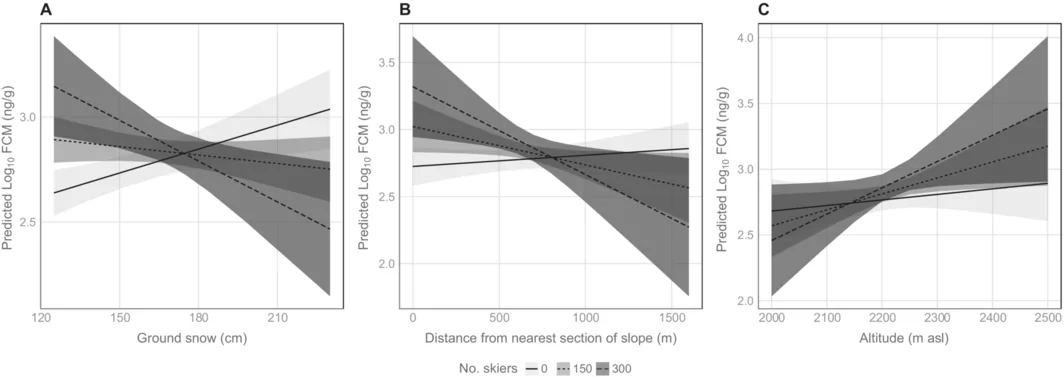
Effect plots of the interaction effects between the number of skiers and various environmental factors—(A) ground snow, (B) distance from ski slope, and (C) altitude—on fecal corticosterone metabolite (FCM) levels in black grouse. CIs displayed are 95% CI. Across the fitted interaction plots shown, predicted log_10_ FCM values ranged approximately from 2.45 to 3.15 for the ground‐snow interaction, from 2.30 to 3.33 for the distance‐to‐slope interaction, and from 2.45 to 3.47 for the altitude interaction, corresponding to maximum differences of roughly 5‐ to 10‐fold on the original FCM scale. asl, above sea level.

We produced spatial predictions of FCM across the study area (Figure 4) using study‐period mean values of the previous day's dynamic covariates—skier passages (143), fresh snowfall (6.7 cm), ground snow depth (150 cm), and temperature (−3.9°C)—to depict typical in‐season conditions. Notably, higher elevations correlate with increased stress levels. Additionally, a significant hotspot of stress is observable around the ski lift arrival area and extends to the upper half of the ski slope, indicating a spatial relationship between ski‐related infrastructure and wildlife stress.

**FIGURE 4:**
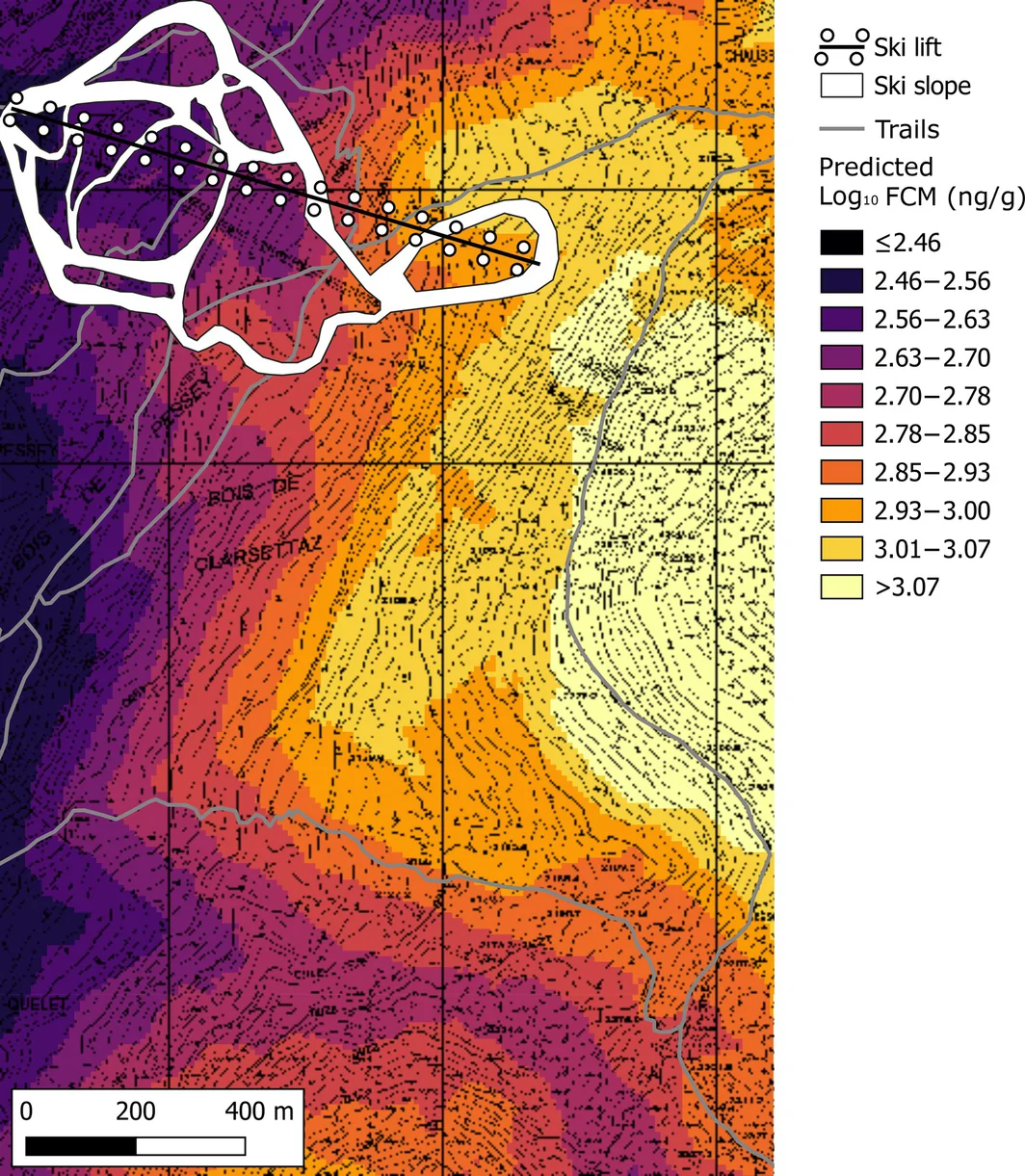
Predicted fecal corticosterone metabolite (FCM) concentrations across the study area under typical in‐season conditions. The map shows model‐predicted log_10_ FCM (ng/g) values and should be interpreted as a relative stress‐marker surface for scenario comparison: Higher values indicate areas where higher FCM levels are predicted under the selected conditions. Dynamic covariates were set to their study‐period means (previous day values over 7 December 2014–23 March 2015): skier passages (143), fresh snowfall (6.7 cm), ground‐snow depth (150 cm), and temperature (−3.9°C). Basemap: Carta Tecnica Regionale (Regione Valle d'Aosta 2005, available at https://geoportale.regione.vda.it/download/ctr/).

To illustrate variability, Figure 5 shows contrasting scenarios using values from the margins of the observed distributions (low vs. high skier activity; shallow vs. deep ground snow). When the number of skiers was kept low (50), increasing the snow depth from 30 to 150 cm showed minimal impact on stress levels (Figure 5A,B). However, with a higher number of skiers (400), the stress response was significantly enhanced across both low and high snow depths, indicating a more pronounced effect of human activity. With 30 cm of snow, the stress was markedly higher compared to low skier conditions (Figure 5C), and this effect was further intensified with 150 cm of snow, concentrating stress particularly around areas with higher human activity such as ski slopes and trails (Figure 5D).

**FIGURE 5:**
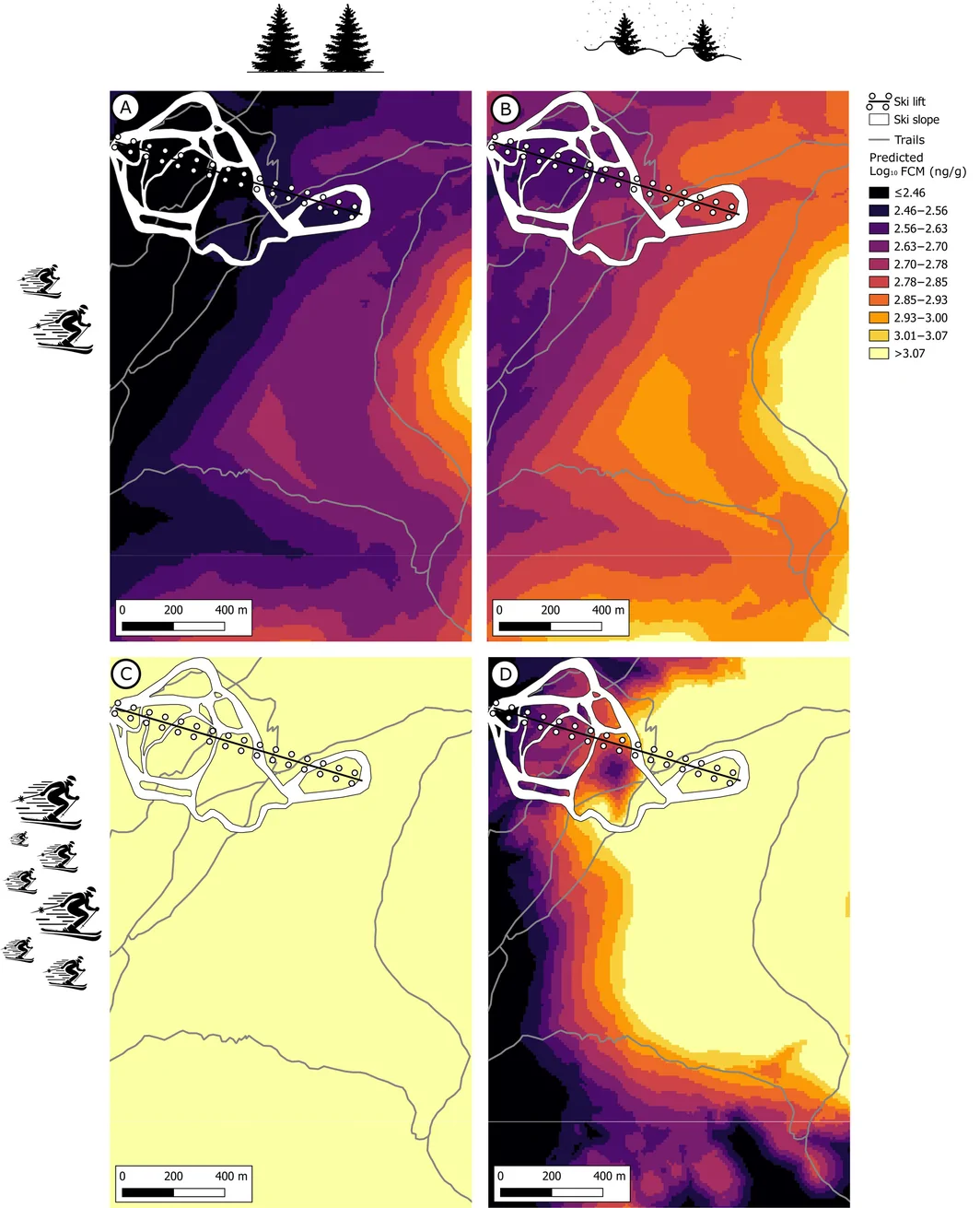
Spatial distribution of predicted fecal corticosterone metabolite (FCM) levels in black grouse under contrasting skier activity and snow‐depth scenarios. The maps show model‐predicted log_10_ FCM (ng/g) values and should be interpreted as relative stress‐marker surfaces within this study: Higher values indicate areas where higher FCM levels are predicted under the specified scenario. Scenarios use representative low and high values from the margins of the observed distributions: (A) 30 cm ground snow and 50 skiers; (B) 150 cm ground snow and 50 skiers; (C) 30 cm ground snow and 400 skiers; (D) 150 cm ground snow and 400 skiers. Figure layout and skier, tree and snow illustrations created by Luca Nelli. Basemap: Carta Tecnica Regionale (Regione Valle d'Aosta 2005, available at https://geoportale.regione.vda.it/download/ctr/).

Our interactive mapping tool, hosted at http://boydorr.gla.ac.uk/lucanelli/blackgrouse_stress/ (Figure 6), allows users to visualize how ski resort activity influences black grouse stress across different combinations of skier presence and environmental conditions. The tool lets users vary these dynamic inputs across their observed ranges and view any combination of conditions between the “typical” map (Figure 4) and the marginal scenarios (Figure 5).

**FIGURE 6:**
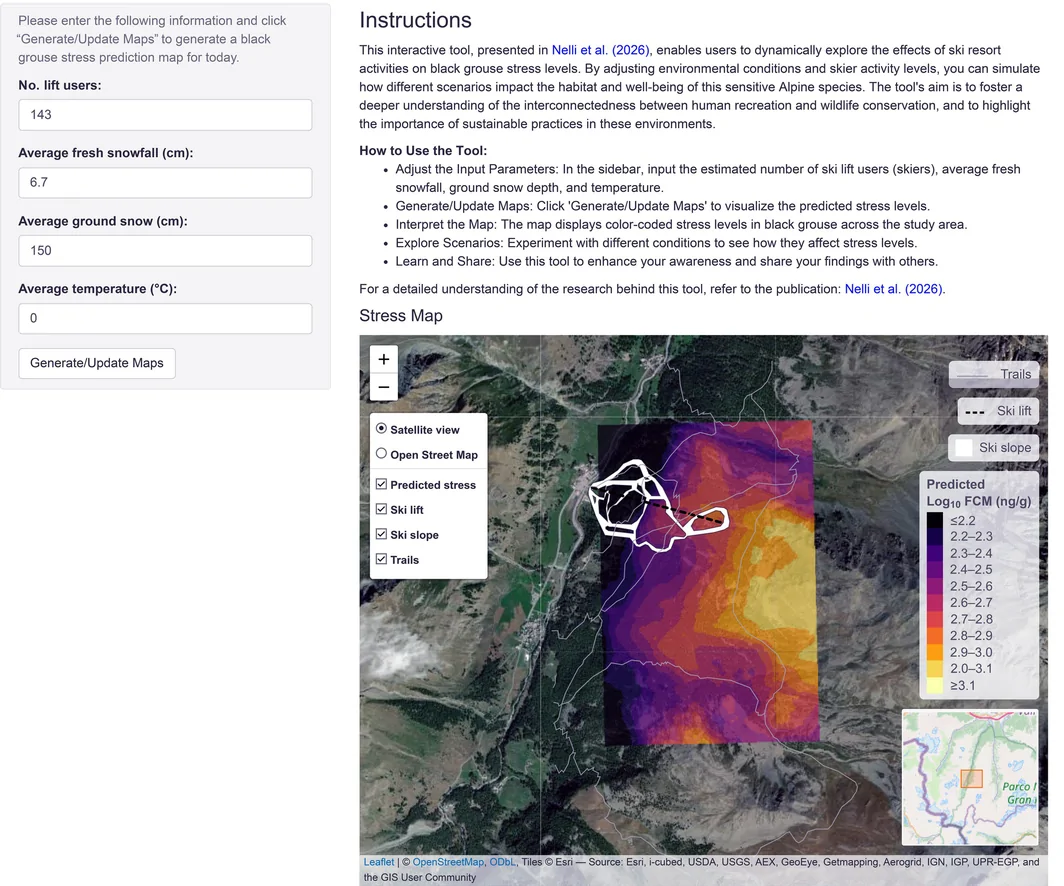
Screenshot of the interactive mapping tool interface. This tool, accessible at http://boydorr.gla.ac.uk/lucanelli/blackgrouse_stress/, allows users to dynamically adjust variables such as skier activity levels and environmental conditions to visualize their impact on black grouse stress levels across different landscapes. FCM, fecal corticosterone metabolite.

## DISCUSSION

In sensitive ecosystems like mountain regions, balancing human recreation with wildlife conservation is a complex challenge (Roseo et al., 2025). Here, we combined noninvasive measurements of physiological stress with mixed‐effects modeling, spatial prediction, and an interactive mapping tool to evaluate and visualize how winter sports are associated with stress responses in black grouse. This provides not only new evidence for the relationship between skiing activity and FCM patterns in our study area but also a practical framework that can be replicated in other systems where comparable data are available.

The absolute values of FCM are not indicative of the magnitude of individual stress because different laboratories, using different extraction methods and EIAs, can produce discordant and therefore incomparable values (Palme, 2019; Palme et al., 2013). However, using these values for relative comparisons across space and time within the same study can be highly informative. Coppes et al. (2018) demonstrated that individual heterogeneity in stress response in capercaillie could overshadow the impacts of environmental stressors unless explicitly accounted for. By incorporating individual identity with FCM analysis, they revealed that individual differences could explain most of the variance observed in FCM levels. Our research supports these findings, indicating significant individual variability in baseline FCM levels among black grouse. This suggests that variation in FCM levels may not solely reflect immediate responses to environmental and anthropogenic conditions but could also be influenced by prior events or inherent factors in individual birds.

In our research, FCM levels increased with higher skier numbers—especially nearer the ski slope—consistent with elevated stress in black grouse during periods of greater ski resort activity (Arlettaz et al., 2007, 2013; Formenti et al., 2015). Although these patterns could also reflect alternative mechanisms—such as habitat selection/avoidance, operational correlates of resort activity (e.g., grooming noise), or snowpack changes (e.g., compaction/hardness) near prepared slopes—we found a clear association between skier activity (particularly near slopes) and higher FCM levels. Similar work on capercaillie *Tetrao urogallus*, a closely related species, has reported associations between ski tourism, habitat use, and adrenocortical activity, highlighting the relevance of recreational pressures on alpine wildlife (Thiel et al., 2008, 2011). In this context, our modeling framework offers a way to move from point‐based FCM measurements to spatially explicit estimates that can be compared across scenarios of human use.

Previous research has shown that FCM concentrations can differ significantly between male and female grouse (Baltic et al., 2005), and that their susceptibility to anthropogenic disturbances may vary due to differences in flushing distances and behaviors (Thiel et al., 2007). In our study, genetic sexing was not attempted: The multiplex panel comprised microsatellite loci for individual identification only and did not include sex‐linked markers, and fecal DNA quantity/quality further constrained sexing. However, it is possible that one sex might avoid the ski area after the ski lift opens, potentially influencing observed FCM levels. Future applications of this approach should, where feasible, incorporate sexing or other individual‐level covariates to refine inference about which demographic groups are most affected. Another limitation of our study is that all fecal samples were collected on days following fresh snowfall, precluding conditions with zero snowfall. This limits our ability to explore how prolonged snow‐free periods affect stress responses in black grouse. Future studies could address this gap, providing insights into their responses to varying snow conditions amid changing climate patterns.

While the observed relationship between altitude and FCM levels may partly reflect the effects of ambient temperature, given the correlation between altitude and temperature in our study area, the clearest pattern from our analysis was the association between FCM levels and human activity. Our models highlight the complexity of human–wildlife interactions, particularly the relationship between skiing activities and FCM variation in black grouse (Arlettaz et al., 2007, 2013, 2015; Thiel et al., 2008; Watson & Moss, 2004). Higher ski resort activity, represented by an increased number of skiers, was associated with higher FCM levels, especially closer to ski infrastructure. Although this pattern is consistent with physiological responses to human disturbance, our observational data cannot identify the behavioral mechanisms involved. Potential pathways include changes in habitat use, vigilance, movement, resting behavior, or exposure to operational features of the ski resort, but these were not measured directly. From a conservation standpoint, our results suggest that managing the spatial and temporal intensity of ski resort activity could help reduce conditions associated with elevated FCM levels in black grouse. In addition to the overall number of skiers, the spatial distribution of their activity appears to play a critical role in influencing black grouse stress levels. The significant interaction between the number of skiers and the distance from the ski slope suggests that the relationship between skier activity and FCM levels weakens with increasing distance from the slope. This pattern provides support for maintaining areas of reduced human activity around ski slopes, as zones farther from high‐activity infrastructure may offer lower disturbance conditions for black grouse, where such lower risk areas are most likely to occur and to inform evidence‐based discussion of buffer‐zone options, rather than relying only on generic or arbitrary rules.

The interplay between ground snow cover and skier numbers further highlights the context dependence of FCM patterns in black grouse. Snow cover can provide important benefits for wintering black grouse, including insulation and concealment from predators (Arlettaz et al., 2007; Bocca et al., 2014), and our model indicates that snow depth modified the relationship between skier activity and FCM levels. Under low skier numbers, predicted FCM levels remained comparatively low across the snow‐depth scenarios considered. As skier numbers increased, however, predicted FCM levels rose, particularly near areas of concentrated human activity such as ski slopes and trails. This indicates that favorable snow conditions do not necessarily remove the association between high skier activity and elevated FCM levels, especially in the parts of the landscape most heavily used for skiing. From a management perspective, these results support considering snow conditions together with the intensity and spatial distribution of human use when assessing disturbance risk. While our study focuses on general skiing activity around prepared slopes, we acknowledge that off‐piste skiing may represent an additional concern for black grouse because it can extend human activity into less predictable and potentially sensitive habitats. This aspect warrants further investigation through targeted studies to better understand its specific impacts and inform appropriate management strategies.

The predictive spatial mapping and interactive tool developed in our study is intended as the applied end point of the framework. By integrating environmental conditions with the number of skiers, the tool allows rapid exploration of predicted FCM patterns under contrasting disturbance scenarios, rather than relying on a single “typical” map. For managers, the maps can help identify where to locate or reinforce refuge areas, how far buffers around infrastructure might extend, and under which combinations of snow conditions and skier activities restrictions may be most beneficial. For resort operators, the tool provides an intuitive way to visualize how patterns of use and prevailing environmental conditions are associated with predicted wildlife stress‐marker levels. In its current form, however, the tool should not be interpreted as a stand‐alone public communication or behavior‐change intervention. Visitor‐facing use would require additional translation of technical model outputs into accessible risk categories, careful message framing, and formal evaluation with end users.

The lack of awareness among recreationists about their influence on wildlife (Gruas et al., 2020) underscores the potential value of tools that make disturbance risk patterns visible and interpretable. However, simply making technical ecological data available is unlikely, by itself, to motivate behavioral change. Effective visitor‐facing communication requires model outputs to be translated into accessible and behaviorally meaningful information, with attention to visual comprehension, cognitive load, message framing, and engagement strategy (Gifford & Comeau, 2011; Harold et al., 2016; Patel et al., 2015). In its current form, our interactive mapping tool is primarily designed for managers, researchers, and resort operators, providing a visual and interactive way to explore how skier activity and environmental conditions are associated with predicted FCM patterns in black grouse. The same framework could support future visitor‐facing communication, but this would require additional translation of model outputs into accessible risk categories, careful message framing, and formal evaluation with end users. This is consistent with recommendations to integrate ecological evidence with social science approaches when seeking to influence visitor understanding and behavior (Bielański et al., 2018; Gruas et al., 2023). The interactive application has not yet been formally piloted with land managers, resort operators, or visitors; informal demonstrations have yielded positive feedback, and structured user testing is planned in future iterations.

By facilitating a dynamic exploration of how different levels of skier activity and environmental conditions are associated with predicted FCM levels in black grouse, this combination of modeling, mapping, and interactive visualization offers a proactive approach to conservation, aligning with Wójcicki et al. (2023)'s call for innovative solutions to mitigate the adverse effects of increased tourism. Although developed for a single alpine resort and species, the same framework can be adapted wherever comparable noninvasive stress data and basic spatial covariates are available, extending its relevance to other taxa, landscapes, and forms of outdoor recreation.

## CONCLUSIONS

We developed and demonstrated a reproducible workflow that links noninvasive measures of physiological stress, mixed‐effects modeling, and spatial prediction to quantify associations between winter sports and wildlife stress markers. Applied to black grouse in an alpine ski resort, this framework highlighted spatial variation in FCM levels associated with skier numbers, proximity to ski infrastructure, and environmental factors such as snow cover and altitude.

Interactive tools that convert model outputs into accessible decision‐support formats have been successfully applied in various contexts, for example, real‐time web applications that inform urban planners by predicting habitat suitability and connectivity for protected species in urban landscapes (Nelli et al., 2022). Building on this approach, our study applies a similar strategy to alpine environments, using an interactive web tool to explore predicted stress under alternative combinations of skier numbers and environmental conditions. This supports both investigation and management of wildlife disturbance by allowing managers and resort operators to compare scenarios and identify areas where targeted mitigation may be most useful.

With further translation of model outputs into nontechnical risk categories and formal user testing, the tool could also be adapted for visitor‐facing communication, for example, through simplified displays in tourist offices or at ski‐lift entrances. However, such use would require dedicated design and evaluation to ensure that the information is understandable, salient, and behaviorally meaningful. More broadly, the underlying workflow is transferable wherever comparable noninvasive stress data and basic spatial covariates are available, offering a practical template for managing human–wildlife interactions in other mountain systems and recreational landscapes.

## AUTHOR CONTRIBUTIONS

**Luca Nelli:** Conceptualization; investigation; data curation; formal analysis; methodology; software; writing—original draft; writing—review and editing. **Lisa Marshall:** Investigation; writing—original draft; writing—review and editing. **Michele Battoraro:** Investigation; writing—original draft. **Geremia Illarietti:** Investigation. **Alberto Meriggi:** Conceptualization; writing—review and editing. **Bruno Bassano:** Conceptualization; writing—review and editing. **Steve Smith:** Investigation; data curation; formal analysis; writing—review and editing. **Jason Matthiopoulos:** Writing—review and editing; methodology. **Rupert Palme:** Investigation; data curation; formal analysis; writing—review and editing.

## CONFLICT OF INTEREST STATEMENT

The authors declare no conflicts of interest.

## Supporting information


Appendix S1.

## Data Availability

Data and code (Nelli et al., 2026) are available in Dryad at https://doi.org/10.5061/dryad.2z34tmq2r.

## References

[eap70336-bib-0001] Antonelli, A. , W. D. Kissling , S. G. A. Flantua , M. A. Bermúdez , A. Mulch , A. N. Muellner‐Riehl , H. Kreft , et al. 2018. “Geological and Climatic Influences on Mountain Biodiversity.” Nature Geoscience 11(10): 718–725.

[eap70336-bib-0002] Arlettaz, R. , S. Nusslé , M. Baltic , P. Vogel , R. Palme , S. Jenni‐Eiermann , P. Patthey , and M. Genoud . 2015. “Disturbance of Wildlife by Outdoor Winter Recreation: Allostatic Stress Response and Altered Activity–Energy Budgets.” Ecological Applications 25(5): 1197–1212. 10.1890/14-1141.1 26485949

[eap70336-bib-0003] Arlettaz, R. , P. Patthey , M. Baltic , T. Leu , M. Schaub , R. Palme , and S. Jenni‐Eiermann . 2007. “Spreading Free‐Riding Snow Sports Represent a Novel Serious Threat for Wildlife.” Proceedings of the Royal Society B: Biological Sciences 274(1614): 1219–1224. 10.1098/rspb.2006.0434 PMC2189568 17341459

[eap70336-bib-0004] Arlettaz, R. , P. Patthey , and V. Braunisch . 2013. “Impacts of Outdoor Winter Recreation on Alpine Wildlife and Mitigation Approaches: A Case Study of the Black Grouse.” In The Impacts of Skiing and Related Winter Recreational Activities on Mountain Environments, edited by C. Rixen and A. Rolando , 137–154. Bussum: Bentham eBooks.

[eap70336-bib-0005] Baltic, M. , S. Jenni‐Eiermann , R. Arlettaz , and R. Palme . 2005. “A Noninvasive Technique to Evaluate Human‐Generated Stress in the Black Grouse.” Annals of the New York Academy of Sciences 1046: 81–95. 10.1196/annals.1343.008 16055845

[eap70336-bib-0006] Barredo, J. I. , A. Mauri , and G. Caudullo . 2020. “Alpine Tundra Contraction under Future Warming Scenarios in Europe.” Atmosphere 11(7): 698.

[eap70336-bib-0007] Bates, D. , M. Maechler , B. Bolker , and S. Walker . 2015. “Fitting Linear Mixed‐Effects Models Using lme4.” Journal of Statistical Software 67(1): 1–48.

[eap70336-bib-0008] Bielański, M. , K. Taczanowska , C. Brandenburg , P. Adamski , and Z. Witkowski . 2018. “Using a Social Science Approach to Study Interactions between Ski Tourers and Wildlife in Mountain Protected Areas.” Mountain Research and Development 38(4): 380–389 310.

[eap70336-bib-0009] Birdlife International . 2015. European Red List of Birds. Luxembourg: Office for Official Publications of the European Communities.

[eap70336-bib-0010] Bocca, M. , E. Caprio , D. Chamberlain , and A. Rolando . 2014. “The Winter Roosting and Diet of Black Grouse *Tetrao tetrix* in the North‐Western Italian Alps.” Journal of Ornithology 155: 183–194.

[eap70336-bib-0011] Büttner, G. , and B. Kosztra . 2018. CLC2018 Technical Guidelines. Vienna: European Environment Agency, European Topic Centre on Urban, Land and Soil Systems.

[eap70336-bib-0013] Caprio, E. , A. Rolando , R. Arlettaz , and D. Chamberlain . 2023. “Anthropogenic Activities and Mountain Birds.” In Ecology and Conservation of Mountain Birds, edited by D. Chamberlain , A. Lehikoinen , and K. Martin , 260–295. Cambridge: Cambridge University Press.

[eap70336-bib-0014] Chang, W. , J. Cheng , J. J. Allaire , Y. Xie , and J. McPherson . 2018. “shiny: Web Application Framework for R.” https://CRAN.R-project.org/package=shiny.

[eap70336-bib-0015] Cheng, J. , B. Karambelkar , and Y. Xie . 2018. “leaflet: Create Interactive Web Maps with the JavaScript ‘Leaflet’.” R package. https://CRAN.R-project.org/package=leaflet.

[eap70336-bib-0016] Cook, C. J. , D. J. Mellor , P. J. Harris , J. R. Ingram , and L. R. Matthews . 2000. “Hands‐On and Hands‐Off Measurement of Stress.” In The Biology of Animal Stress, edited by G. P. Moberg and J. A. Mench , 123–146. Wallingford: CABI Publishing.

[eap70336-bib-0017] Coppes, J. , J.‐L. Kämmerle , M. Willert , A. Kohnen , R. Palme , and V. Braunisch . 2018. “The Importance of Individual Heterogeneity for Interpreting Faecal Glucocorticoid Metabolite Levels in Wildlife Studies.” Journal of Applied Ecology 55(4): 2043–2054.

[eap70336-bib-0018] DeWoody, J. A. , J. Schupp , L. Kenefic , J. Busch , L. Murfitt , and P. Keim . 2004. “Universal Method for Producing ROX‐Labeled Size Standards Suitable for Automated Genotyping.” BioTechniques 37(3): 348, 350, 352. 10.2144/04373BM02 15470886

[eap70336-bib-0019] Dirnböck, T. , F. Essl , and W. Rabitsch . 2011. “Disproportional Risk for Habitat Loss of High‐Altitude Endemic Species under Climate Change.” Global Change Biology 17(2): 990–996.

[eap70336-bib-0020] European Commission . 2009. “Directive 2009/147/EC of the European Parliament and of the Council of 30 November 2009 on the Conservation of Wild Birds.” https://eur-lexeuropaeu/legal-content/EN/TXT/?uri=CELEX:32009L0147.

[eap70336-bib-0021] Formenti, N. V. R. , R. Bionda , N. Ferrari , T. Trogu , P. Lanfranchi , and R. Palme . 2015. “Increased Hormonal Stress Reactions Induced in an Alpine Black Grouse (*Tetrao tetrix*) Population by Winter Sports.” Journal of Ornithology 156: 317–321.

[eap70336-bib-0022] Gifford, R. , and L. A. Comeau . 2011. “Message Framing Influences Perceived Climate Change Competence, Engagement, and Behavioral Intentions.” Global Environmental Change 21: 1301–1307.

[eap70336-bib-0023] Gruas, L. , A. Loison , M. Ba , and C. Perrin‐Malterre . 2023. ““If we Really Disturbed them, They Would Leave”: Mountain Sports Participants and Wildlife Disturbance in the Northern French Alps.” Journal of Outdoor Recreation and Tourism 42: 100610.

[eap70336-bib-0024] Gruas, L. , C. Perrin‐Malterre , and A. Loison . 2020. “Aware or Not Aware? A Literature Review Reveals the Dearth of Evidence on Recreationists Awareness of Wildlife Disturbance.” Wildlife Biology 2020(4): wlb.00713.

[eap70336-bib-0025] Harold, J. , I. Lorenzoni , T. F. Shipley , and K. R. Coventry . 2016. “Cognitive and Psychological Science Insights to Improve Climate Change Data Visualization.” Nature Climate Change 6: 1080–1089.

[eap70336-bib-0026] Hijmans, R. J. 2020. “Terra: Spatial Data Analysis.” CRAN: Contributed Packages, The R Foundation. 10.32614/cran.package.terra.

[eap70336-bib-0027] Ludwig, G. X. , R. V. Alatalo , P. Helle , H. Lindén , J. Lindström , and H. Siitari . 2006. “Short‐ and Long‐Term Population Dynamical Consequences of Asymmetric Climate Change in Black Grouse.” Proceedings of the Royal Society B: Biological Sciences 273(1597): 2009–2016. 10.1098/rspb.2006.3538 PMC1635476 16846907

[eap70336-bib-0028] Möstl, E. , and R. Palme . 2002. “Hormones as Indicators of Stress.” Domestic Animal Endocrinology 23(1–2): 67–74. 10.1016/s0739-7240(02)00146-7 12142227

[eap70336-bib-0029] Nelli, L. 2024. “Color Quest: An Interactive Tool for Exploring Color Palettes and Enhancing Accessibility in Data Visualization.” PLoS One 19(3): e0290923. 10.1371/journal.pone.0290923 PMC10950247 38502671

[eap70336-bib-0030] Nelli, L. , L. Marshall , M. Battoraro , G. Illarietti , A. Meriggi , B. Bassano , J. Matthiopoulos , S. Smith , and R. Palme . 2026. “Data and Code from: Skiers Footprint on Wildlife Stress: Tracing the Impact of Winter Sports on Black Grouse with Interactive Mapping.” Dryad. 10.5061/dryad.2z34tmq2r.

[eap70336-bib-0031] Nelli, L. , M. Murru , and A. Meriggi . 2016. “Effects of Density on Lek‐Site Selection by Black Grouse (*Tetrao tetrix*) in the Alps.” Bird Study 63: 187–195.

[eap70336-bib-0032] Nelli, L. , B. Schehl , R. A. Stewart , C. Scott , S. Ferguson , S. MacMillan , and D. J. McCafferty . 2022. “Predicting Habitat Suitability and Connectivity for Management and Conservation of Urban Wildlife: A Real‐Time Web Application for Grassland Water Voles.” Journal of Applied Ecology 59(4): 1072–1085.

[eap70336-bib-0033] Palme, R. 2019. “Non‐Invasive Measurement of Glucocorticoids: Advances and Problems.” Physiology & Behavior 199: 229–243. 10.1016/j.physbeh.2018.11.021 30468744

[eap70336-bib-0034] Palme, R. , C. Touma , N. Arias , M. F. Dominchin , and M. Lepschy . 2013. “Steroid Extraction: Get the Best Out of Faecal Samples.” Wiener Tierärztliche Monatsschrift 100: 238–246.

[eap70336-bib-0035] Patel, M. S. , D. A. Asch , and K. G. Volpp . 2015. “Wearable Devices as Facilitators, Not Drivers, of Health Behavior Change.” JAMA 313(5): 459–460. 10.1001/jama.2014.14781 25569175

[eap70336-bib-0036] Patthey, P. , N. Signorell , L. Rotelli , and R. Arlettaz . 2012. “Vegetation Structural and Compositional Heterogeneity as a Key Feature in Alpine Black Grouse Microhabitat Selection: Conservation Management Implications.” European Journal of Wildlife Research 58(1): 59–70.

[eap70336-bib-0037] Pearce‐Higgins, J. W. , and K. Martin . 2023. “Climate Change Impacts on Mountain Birds.” In Ecology and Conservation of Mountain Birds, edited by D. Chamberlain , A. Lehikoinen , and K. Martin , 215–259. Cambridge: Cambridge University Press.

[eap70336-bib-0038] Pebesma, E. 2018. “Simple Features for R: Standardized Support for Spatial Vector Data.” The R Journal 10(1): 439–446.

[eap70336-bib-0039] Piertney, S. B. , and J. Höglund . 2001. “Polymorphic Microsatellite DNA Markers in Black Grouse (*Tetrao tetrix*).” Molecular Ecology Notes 1(4): 303–304.

[eap70336-bib-0040] R Development Core Team . 2024. R: A Language and Environment for Statistical Computing. Vienna: R Foundation for Statistical Computing.

[eap70336-bib-0041] Rettenbacher, S. , E. Möstl , R. Hackl , K. Ghareeb , and R. Palme . 2004. “Measurement of Corticosterone Metabolites in Chicken Droppings.” British Poultry Science 45(5): 704–711. 10.1080/00071660400006156 15623226

[eap70336-bib-0042] Rogerson, P. A. 2001. Statistical Methods for Geography. London: Sage.

[eap70336-bib-0043] Roseo, F. , C. Celada , and M. Brambilla . 2025. “Ski Resorts Threaten Climate Refugia for High‐Elevation Biodiversity under Current and Future Conditions in the Alps.” Biological Conservation 301: 110890.

[eap70336-bib-0044] Rumpf, S. B. , M. Gravey , O. Brönnimann , M. Luoto , C. Cianfrani , G. Mariethoz , and A. Guisan . 2022. “From White to Green: Snow Cover Loss and Increased Vegetation Productivity in the European Alps.” Science 376(6597): 1119–1122. 10.1126/science.abn6697 35653482

[eap70336-bib-0045] Sapolsky, R. M. , L. M. Romero , and A. U. Munck . 2000. “How Do Glucocorticoids Influence Stress Responses? Integrating Permissive, Suppressive, Stimulatory, and Preparative Actions.” Endocrine Reviews 21(1): 55–89. 10.1210/edrv.21.1.0389 10696570

[eap70336-bib-0046] Sato, C. F. , J. T. Wood , and D. B. Lindenmayer . 2013. “The Effects of Winter Recreation on Alpine and Subalpine Fauna: A Systematic Review and Meta‐Analysis.” PLoS One 8(5): e64282. 10.1371/journal.pone.0064282 PMC3655029 23691190

[eap70336-bib-0047] Schweiger, A. K. , U. Nopp‐Mayr , and M. Zohmann . 2012. “Small‐Scale Habitat Use of Black Grouse (*Tetrao tetrix* L.) and Rock Ptarmigan (*Lagopus muta helvetica* Thienemann) in the Austrian Alps.” European Journal of Wildlife Research 58(1): 35–45.

[eap70336-bib-0048] Segelbacher, G. , R. J. Paxton , G. Steinbrück , P. Trontelj , and I. Storch . 2000. “Characterization of Microsatellites in Capercaillie *Tetrao urogallus* (AVES).” Molecular Ecology 9(11): 1934–1935. 10.1046/j.1365-294x.2000.0090111934.x 11091338

[eap70336-bib-0049] Storch, I. 2007. “Conservation Status of Grouse Worldwide: An Update.” Wildlife Biology 13: 5–12.

[eap70336-bib-0050] Tarquini, S. , I. Isola , M. Favalli , A. Battistini , and G. T. Dotta . 2023. A Digital Elevation Model of Italy with a 10 Meters Cell Size (Version 1.1), Vol. 1, 1–2. Roma: Istituto Nazionale di Geofisica e Vulcanologia (INGV).

[eap70336-bib-0051] Thiel, D. , S. Jenni‐Eiermann , V. Braunisch , R. Palme , and L. Jenni . 2008. “Ski Tourism Affects Habitat Use and Evokes a Physiological Stress Response in Capercaillie *Tetrao urogallus*: A New Methodological Approach.” Journal of Applied Ecology 45(3): 845–853.

[eap70336-bib-0052] Thiel, D. , S. Jenni‐Eiermann , and R. Palme . 2005. “Measuring Corticosterone Metabolites in Droppings of Capercaillies (*Tetrao urogallus*).” Annals of the New York Academy of Sciences 1046: 96–108. 10.1196/annals.1343.009 16055846

[eap70336-bib-0053] Thiel, D. , S. Jenni‐Eiermann , R. Palme , and L. Jenni . 2011. “Winter Tourism Increases Stress Hormone Levels in the Capercaillie *Tetrao urogallus* .” Ibis 153(1): 122–133.

[eap70336-bib-0054] Thiel, D. , E. Ménoni , J.‐F. Brenot , and L. Jenni . 2007. “Effects of Recreation and Hunting on Flushing Distance of Capercaillie.” The Journal of Wildlife Management 71(6): 1784–1792.

[eap70336-bib-0055] Tsuyuzaki, S. 2002. “Vegetation Development Patterns on Skislopes in Lowland Hokkaido, Northern Japan.” Biological Conservation 108(2): 239–246.

[eap70336-bib-0056] Urbanska, K. M. 1997. “Restoration Ecology Research above the Timberline: Colonization of Safety Islands on a Machine‐Graded Alpine Ski Run.” Biodiversity and Conservation 6(12): 1655–1670.

[eap70336-bib-0057] Watson, A. , and R. Moss . 2004. “Impacts of Ski‐Development on Ptarmigan (*Lagopus mutus*) at Cairn Gorm, Scotland.” Biological Conservation 116(2): 267–275.

[eap70336-bib-0058] Weber, K. , F. Hoelzl , J. S. Cornils , S. Smith , C. Bieber , B. Balint , and T. Ruf . 2018. “Multiple Paternity in a Population of Free‐Living Edible Dormice (*Glis glis*).” Mammalian Biology 93: 45–50.

[eap70336-bib-0059] Wingfield, J. C. , D. L. Maney , C. W. Beruner , J. D. Jacobs , S. Lynn , M. Ramenofsky , and R. D. Richardson . 2015. “Ecological Bases of Hormone—Behavior Interactions: The “Emergency Life History Stage”.” American Zoologist 38(1): 191–206.

[eap70336-bib-0060] Wójcicki, A. , W. Świtlik , and D. Dobrowolska . 2023. “The Need for Wildlife Research and Improved Management of Protected Areas in the Face of Increased Nature‐Based Tourism.” Environmental Protection and Natural Resources 34(3): 20–28.

