# Supplementary material for: Skiers' footprint on wildlife stress: Tracing the impact of winter sports on black grouse with interactive mapping
Source: Ecol Appl. 2026 Oct 21;36(7):e70336. doi: 10.1002/eap.70336 (PMC13685650; doi:10.1002/eap.70336)
Supplement: Supplementary file 1 [file EAP-36-e70336-s001.pdf]

Appendix S1

Skiers' footprint on wildlife stress: Tracing the impact of winter sports on black grouse with interactive mapping

Luca Nelli, Lisa Marshall, Michele Battoraro, Geremia Illarietti, Alberto Meriggi, Bruno Bassano, Jason Matthiopoulos, Steve Smith, and Rupert Palme

Ecological Applications

Model-selection transparency for retained interaction terms

This appendix reports the ΔAIC values supporting the interaction terms retained in the final mixed-effects model. For each retained interaction, ΔAIC was calculated as AICreduced − AICfinal, where the reduced model excludes the focal interaction while retaining the corresponding lower-order main effects. Positive ΔAIC values indicate that removing the interaction worsened model fit relative to the final model.

Table S1. ΔAIC values for interaction terms retained in the final model.

Interaction term	AIC final model	AIC reduced model	ΔAIC
N_SKI × GSNOW	193.383	196.671	3.288
N_SKI × D_slope	193.383	201.443	8.060
N_SKI × D_larch	193.383	197.374	3.991
N_SKI × ALT	193.383	198.162	4.779

Note: ΔAIC was calculated as AICreduced − AICfinal. All retained interaction terms had ΔAIC > 2, supporting their inclusion under the interaction-retention criterion described in the Methods.
